# Statin Consumption and Appealing Colors: Exploring Statin-Related Injuries for Children Under the Age of Three Years

**DOI:** 10.7759/cureus.73520

**Published:** 2024-11-12

**Authors:** Allison Brown, Vishveshvar Ramkumar, Aditi Patel, David Kang, Jedidiah Lim, Samreen Shah, Hassan Y Ebrahim, Zakaria Y Abd Elmageed

**Affiliations:** 1 Department of Biomedical Sciences, Discipline of Pharmacology, Edward Via College of Osteopathic Medicine, Monroe, USA

**Keywords:** children health, drug safety, neiss, prescription safety, statins

## Abstract

Introduction: Statins are frequently prescribed to lower the risk of atherosclerosis and cardiovascular-related diseases. While statins are considered safe, there are occasional accidental overdoses in children that warrant concern for how to protect children from unintended consumption. We aimed to determine which statins were more prone to injury, characterize the injury types commonly seen for each statin, assess the age at which statin-related injuries were most frequent, and compare statin-related injuries among genders.

Methods: We accessed the National Electronic Injury Surveillance System (NEISS) database to collect hospital cases of drug-related injuries among children that occurred between 2013-2022. Out of these cases, subjects for this study were selected based on the inclusion criteria of statin-related injury. Additionally, we used disposition codes to identify the outcomes for each statin and children under three years of age. Descriptive statistics were utilized to display the frequency of disposition codes corresponding to specific statins and statin-related injuries by age. A regression analysis was then conducted to create a trend line showing the incidence of statin-related injury among males.

Results: From 2013 to 2022, there were 81 statin-related injuries. Across the different statins, atorvastatin had the highest incidence of injuries among children under three years old (n=51), with a hospitalization rate of 39.22%. However, atorvastatin had the lowest hospitalization rate compared to other medications, such as rosuvastatin (67.0%) and simvastatin (47.0%). Hospitalization criteria were based on the disposition code 4: treated and admitted to the hospital. When comparing statin-related injuries in terms of age, we specifically found that atorvastatin-related consumption increased exponentially from nine months (n=1) to its highest occurrence at 24 months (n=16) with a percent change of 15%. The elevated occurrence at 24 months suggests that some developmental milestones in infants may make children more susceptible to atorvastatin-related injury. Additionally, a notable absence of statin-related injuries was identified after 24 months, followed by a recurrence at 36 months of age (n=8). When comparing statin-related injuries in terms of female and male children under three years, a notable finding was the continuous increase in male injuries from 2013-2021. The increase is significant in 2021, where there were a total of nine cases; two were female, and seven were male. The data showed a greater number of male cases (55.8%). The data also showed a rise in male visits to the emergency department between 2018 and 2021, possibly due to COVID-19. To explain, more children were at home with their parents/caretakers, which could have been the reason for the increase in accidental ingestion of statins.

Conclusions: Producing statins in colors appealing to children can increase the incidence of accidental consumption. This risk peaks till the age of two years, coinciding with the completion of the oral fixation developmental milestone. To address this issue, Electronic Health Records (EHR) prompts can assist physicians in taking a more proactive approach to prescription safety during their discussions with patients to create a safer environment for children.

## Introduction

Statins have emerged as a cornerstone in preventing and treating various cardiovascular diseases (CVDs), with an estimated 92 million individuals in the United States of America treated with statins for cholesterol management in 2019 [1]. Statins, also known as β-Hydroxy β-methylglutaryl-CoA (HMG-CoA) reductase inhibitors, exert their primary effect by inhibiting HMG-CoA reductase enzyme responsible for cholesterol biosynthesis in the liver [2]. By lowering circulating low-density lipoprotein cholesterol (LDL-C) levels, statins mitigate a pivotal risk factor for atherosclerosis and numerous cardiovascular ailments, including coronary artery disease (CAD) and stroke [2]. Moreover, these agents exhibit pleiotropic effects beyond cholesterol reduction-encompassing anti-inflammatory, antioxidative, and endothelial function improvement properties, further contributing to their cardioprotective attributes [3-4]. 

While statins are generally considered safe and effective when used appropriately, instances of accidental or intentional overdose in children raise concerns regarding potential adverse effects and appropriate management strategies [5]. Statin overdose in children can occur due to unintentional ingestion of medication, medication errors, or deliberate self-harm. The consequences of statin overdose in pediatric patients may range from mild gastrointestinal symptoms (constipation, diarrhea, indigestion) to more severe manifestations, such as myopathy, rhabdomyolysis, and hepatotoxicity [6]. Given the lack of specific antidotes for statin overdose, management primarily focuses on supportive measures and symptomatic treatment. This article aims to provide insight into child morbidity associated with parental and guardian statin use.

## Materials and methods

Data was sourced from the National Electronic Injury Surveillance System (NEISS), a national database that reports anonymous hospital cases of product-related injuries involving children and adults of all ages in the United States. The source of this data is both private and non-private emergency hospitals. The available data was extracted from 2013 to 2022, including drug poisoning to children under the age of three years and diagnosis of aspiration/ingestion (coded as 41/42 in the database, n = 10841). This data was further screened by looking into the “narrative” of each case (n=81) and finding keywords that end with 'statin' (drugs include simvastatin, atorvastatin, pravastatin, lovastatin, and rosuvastatin).

Data for disposition or outcome of the patient was further examined by using NEISS disposition codes as following, 1= treated and released or released without treatment, 2 = treated and transferred to another hospital, 4 = treated and admitted for hospitalization of the same facility, 5 = held for observation, which includes admission for observation, 6 = left without being seen, left AMA, 8 = fatality, includes dead on arrival, and 9 = not recorded. Disposition codes for each statin-related injury case were then isolated from the sourced data and utilized to construct a bar graph demonstrating the prevalence of specific outcomes related to each statin drug.

Additionally, another bar graph was constructed comparing statin-related injuries among different age groups to identify the ages at which specific statins were more likely to cause injury (n = 81). Furthermore, the incidence of statin-related injuries by gender was compared (n = 81). The gender data was collected from 2013 to 2022 for children under the age of three years. The database was accessed in 2024.

## Results

Statins that were more prone to injury in specific age groups were found. Identifying this information helped us to locate patterns regarding which statins were more frequently associated with injury across age groups up to three years of age. These patterns were represented through a trend line to demonstrate changes in the prevalence of specific statin-related injuries by age. For instance, if a specific statin was more frequently reported for injury across all groups, this may suggest that properties regarding the drug, such as color, size, and accessibility, may increase its susceptibility to injury.

**Figure 1:**
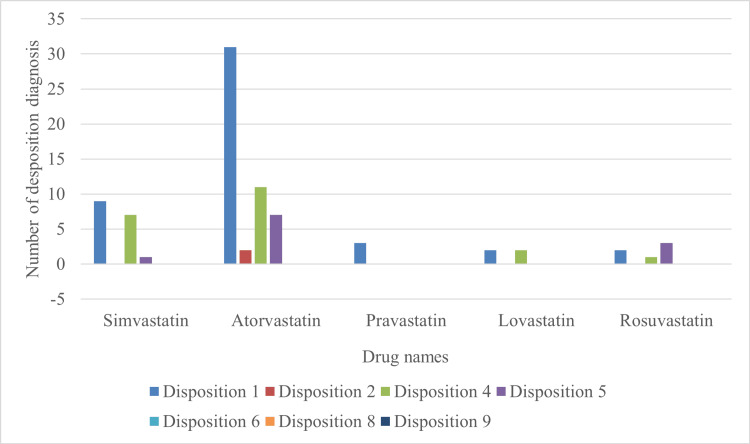
The number of cases per disposition class was respective for the five most commonly used statins. Data is for children under the age of three years within the time period of 2013 and 2022. Disposition codes from the NEISS are presented in Table 1.

Based on NEISS data from 2013 to 2022, we utilized a comparative analysis through a bar graph to determine which statins were more likely associated with injuries. This information helped pinpoint which statins require a greater emphasis on intervention strategies. In Figure 1, 81 cases of statin-related injuries were reported to the NEISS database between 2013 and 2022. Atorvastatin had 51 cases, with 18/50 (36%) admitted to the hospital. Simvastatin had 17 cases of injury, with 8/17 (47%) cases admitted to the hospital. Rosuvastatin with six cases with 4/6 (67%) patients admitted to the hospital. Lovastatin had four cases, and half were admitted to the hospital (50%). The final drug analyzed was pravastatin with three total cases and no hospital admittances (0%). The hospital admission criteria refer to dispositions 4 and 5 from Table 1. No fatalities due to statin overdose were reported.

**Table 1:** The NEISS disposition code used for values is found in Figure 1. NEISS: National Electronic Injury Surveillance System; ED: Emergency Department

Disposition	Code
Treated and released, or examined and released without treatment (also includes transfers for treatment to another department of the same facility without admission)	1
Treated and transferred to another hospital	2
Treated and admitted for hospitalization (within same facility)	4
Held for observation (included admitted for observation)	5
Left without being seen, left against medical advice, left without treatment, loped	6
Fatality, included dead on arrival (“DOA”), died in the ED, and died after admission	8
Not recorded	9

Atorvastatin demonstrated the highest overall incidence of reported cases (n=51); however, its hospitalization rate was lower than rosuvastatin and simvastatin. The case number was calculated by going through the patient's present illness (HPI) and highlighting keywords such as ‘statin’. This helped narrow down the data of incidental ingestion to statin-specific drugs, specifically atorvastatin, for the highest overall count. Most statin injuries were related to atorvastatin consumption and were classified under dispositions 1 and 4. Based on the disposition codes depicted in Table 1, disposition 1-related injuries denote non-serious injuries where children were treated and discharged or discharged without treatment. Furthermore, disposition 4-related injuries indicate more severe injuries with children who required treatment and were admitted for hospitalization. Compared to other statins, pravastatin consumption occurred the least, with only three reported cases of disposition 1-related injury.

**Figure 2:**
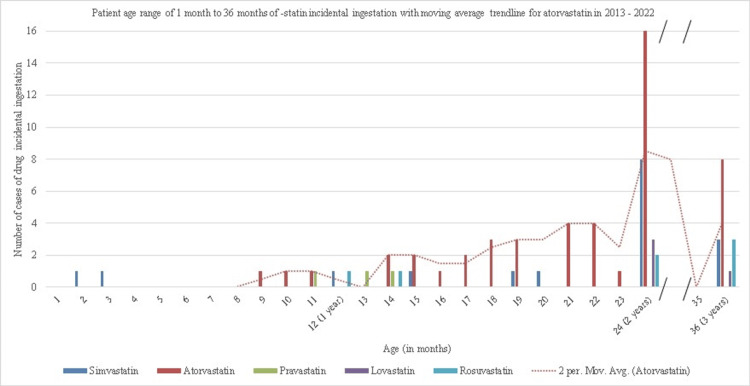
A representation of the number of cases per statin for an age range between one month to 36 months Data is from children ranging from one month to 36 months (three years) of age from 2013 to 2022. There is a broken axis from 25 months to 35 months due to no incidents of children in that age. The data has been represented as number of cases at certain ages (1-36 months).

In Figure 2, the highest prevalence of statin-related injury at one and three months of age occurred from atorvastatin consumption, as indicated. It suggests that there may be a characteristic of atorvastatin contributing to its frequent accidental consumption in children. Specifically, the trend line demonstrated that atorvastatin-related consumption increased exponentially from nine months (n = 1) to its highest occurrence at 24 months (n = 16) with a percent change of 15%. The elevated occurrence at 24 months suggests that some developmental milestones in infants may make children more susceptible to atorvastatin-related injury. Additionally, a notable absence of statin-related injuries was identified after 24 months, followed by a recurrence at 36 months of age (n = 8). This rebound in statin-related injuries may suggest that after 24 months, children are less prone to drug-related injuries.

**Figure 3:**
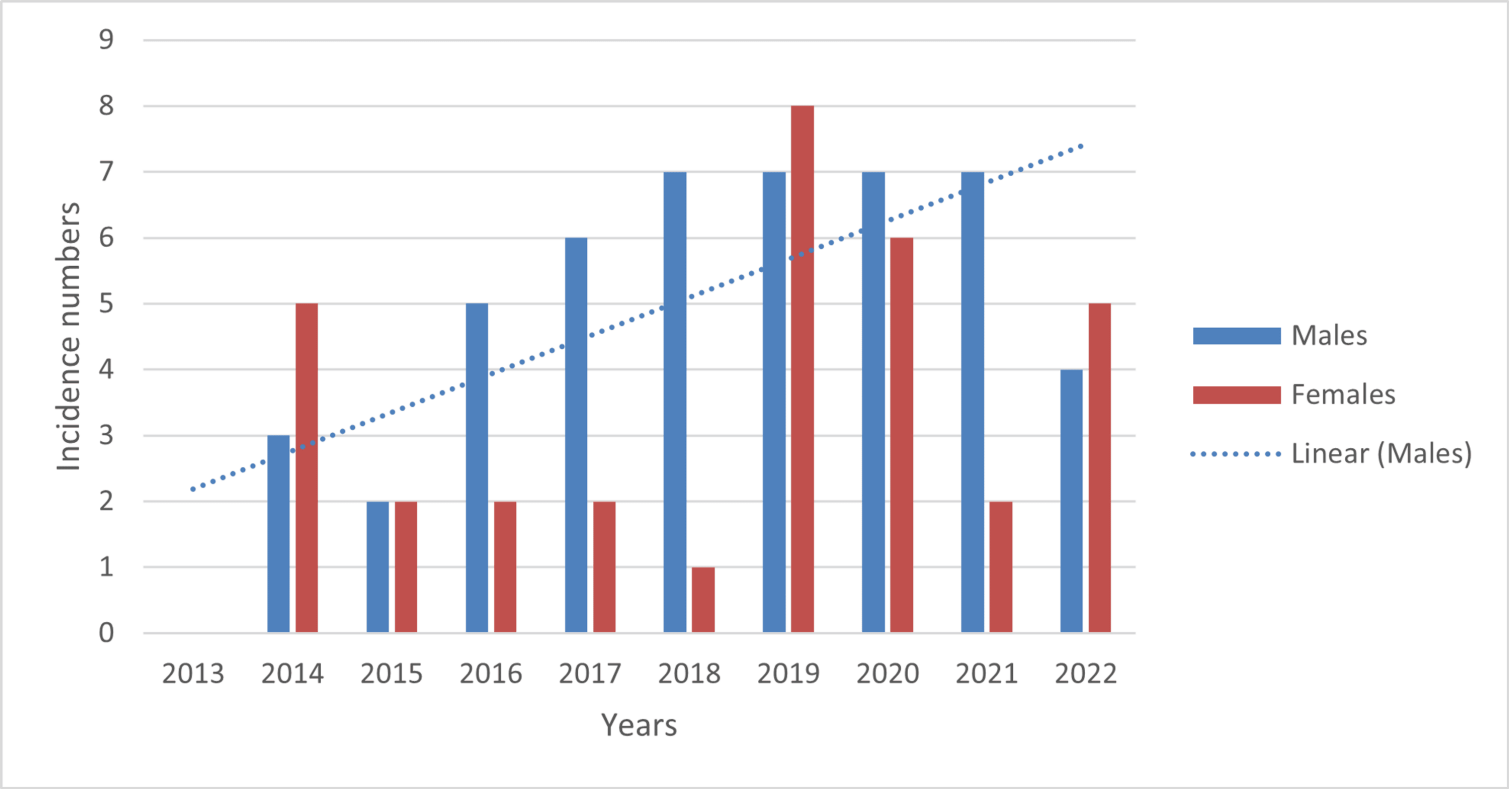
A comparison of the incidence of statin related accidental ingestions of children under the age of three years The linear trend line shows the increase in cases in males specifically and how this trend remains higher compared to female incident rates. Of note, the year of 2013 did not have any statin incidental ingestion cases. The data has been represented as incidence numbers over 10 years (2013 to 2022).

The data shown in Figure 3 demonstrated the male-to-female ratio of incidence numbers from each reported year. In 2013, there were no statin-related injuries for children under three years of age reported to the NEISS database. In 2014, there were a total of eight cases; five (62.5%) were females and three (37.5%) were males. The data showed a greater number (by 0.25%) of female cases. In 2015, there were a total of four cases; two were females and two were males (50% each). The data showed no difference in the amount of incidental stain ingestion between the two sexes.

In 2016, there were a total of seven cases; two (28.6%) were females and five (71.4%) were males. The data showed a greater number of male cases by 42.4%. In 2017, there were a total of eight cases; two (25%) were females and six (75%) were males. The data showed a greater number of male cases by 50%. In 2018, there were a total of eight cases; one (12.5%) was female and seven (87.5) were males. The data showed a greater number of male cases by 75%. In 2019, there were a total of 15 cases; eight (53.3%) were females and seven (46.7%) were males. The data showed a greater number of female cases by 6.3%. In 2020, there were a total of 13 cases; six (46.2%) were females and seven (53.8%) were males. The data showed a greater number of male cases by 8%. In 2021, there were a total of nine cases; two (22.2%) were females and seven (77.8%) were males. The data showed a greater number of male cases by 55.8%. In 2022, there were a total of nine cases; five (55.6%) were females and four (44.4%) were males. The data showed a greater number of female cases by 1.2%. A trend line was added, showing an increase in male cases between 2014-2022. The NEISS databases showed there were greater overall cases of accidental drug ingestion after 2014 and showed a rise in male visits to the emergency department between 2018- 2021, possibly due to the COVID-19 pandemic. To explain, more children were at home with their parents/caretakers, which could have been the reason for the increase in accidental ingestion of statins.

## Discussion

The NEISS review identified that 13.58% of pediatric statins accidental ingestion cases were held for observation in the hospital, 25.93% of cases were treated and admitted to the hospital, and 2.47% of cases were treated and transferred to a different hospital. In total, 41.98% of incidental statin ingestion in those under the age of three were “serious.” A review of adverse drug reactions of statins in children and adolescents, using the World Health Organization's global database of individual safety data reports (WHO ISDR), found comparable results. The WHO ISDR review highlighted that 14.1% of adverse drug reactions were accidental or intentional exposure, 14.5% were overdoses, and 3.5% were off-label use - of these reports, 42.8% of reports had “serious” health effects [7].

Atorvastatin produced the highest prevalence of statin-related injury across all age groups and statins; specifically, atorvastatin-related incidence steadily increases between 13 months of age until it spikes at 24 months of age. However, simvastatin produced severe health effects, including cardiovascular effects and hepatic abnormalities, at a rate of 47.09% when compared to all simvastatin ingestion incidences, while atorvastatin produced severe health effects at a rate of 39.22%. Severe health effects include constipation, diarrhea, dehydration secondary to diarrhea, and headaches. Lastly, rosuvastatin produced the highest percentage of hospitalizations at 67%. Adverse health effects of all drugs include, but are not limited to diarrhea, nausea, and fatigue - producing dehydration and overall weakness. Although statin effects are considered mild due to their low bioavailability, more serious and rare complications of statin toxicity include the development of type two diabetes and neurological symptoms [8]. Bioavailability correlates with increased hospitalization with rosuvastatin; rosuvastatin has the highest bioavailability at 20% compared to simvastatin (5%) and atorvastatin (14%) [9]. Considering the young age of the patients and that the pancreas has not fully matured, it is important to follow the patients long-term to obtain further insight into the possible toxic effects of statin ingestion in children under three years of age [10].

Based on the NEISS review, simvastatin has the highest frequency of incidental ingestion among children under the age of three, requiring hospitalization. Because it is not the most prescribed statin, psychological and physiological factors likely play a role in its incidental ingestion [1]. An association between color and consumption of media, food, and medication has been observed in previous studies [11,12]. In a systematic review of pill color preference in children, the study reported that in addition to other contributing factors, children preferred bright colors, but the colors red and pink were liked best among children [13]. It is speculated that early in life, children associate the color red with sweetness [14]. A correlation between the color red, sweetness, and candy can be seen with the incidence of ingestion of simvastatin in children. In addition, pravastatin, lovastatin, and rosuvastatin are commonly manufactured in bright green, blue, and red-orange colors, respectively. Although less common, atorvastatin is produced by a minority of manufacturers in a bright yellow color. The variation in accidental consumption rates between pravastatin and atorvastatin may hint at the distinct physical differences in the properties of these statin medications as a factor in their potential for being consumed inappropriately.

Beyond color association, there are physiological factors that are associated with incidental ingestion of statins in children. Specifically, age-specific incidence rates coincide with developmental milestones for children. Starting at ages six to nine months, children begin to bring items to their mouth and explore things orally; consequently, they are more likely to ingest items, as displayed by the increase in incidence at that age range [15,16]. Furthermore, figure three shows a dramatic increase around two years of age. The developmental milestones that correlate with this age range include parallel play, standing on tiptoes, and greater hand-mouth/eye coordination, all skills that make it easier for a child to ingest items [15].

In addition, studies have shown differences in age-related milestones based on sex. A study reviewing sex-specific milestones in children between birth and the age of six saw that gender-neutral developmental scales could lead to decreased diagnosing of females with developmental delay [17]. Because females proceed with males in most developmental milestones, females with developmental delays, when reviewed on a gender-neutral scale, can go undiagnosed. Resultantly, spikes of ingestion incidences could be related to different genders developing parallel play at different time intervals - with females developing it first and males following. Although females and males acquire developmental goals at different intervals, both genders develop to the same level which can be observed in Figure 3 as there is no significant difference in male and female incidental ingestion [18]. Figure 3 also aligns the evidence that female and male rates of drug overdose vary by year and, on average, have no statistically significant difference.

Despite the increased incidence of statin ingestion and overdose being correlated to psychological and physiological factors - such as color perception and development milestones in children, this review has limitations. First, the number of ingestion incidences for the 10-year review period totaled 81; because of the small sample size, the review is limited in looking at correlations that expand beyond gender, psychological preference, and physiological development. The database itself also limits the scope of the study. Due to variances in record keeping between hospitals, the gap in data between 24 months of age and 36 months of age could stem from age being tracked in years or due to no incidences during that age range. Furthermore, the database does not allow for the evaluation of education and socioeconomic factors, both of which play a role in medication and home safety [19]. In all, statin overdose may continue to increase as the rates of hypercholesterolemia, obesity, and heart disease continue to increase. It is important to increase awareness of child overdose and toxicity as well as increase pill safety and education among parents [20].

The number of statin overdoses can be reduced through the implementation of secure storage solutions for prescription pills and more involved discussions with physicians on medication safety. EHR prompts for discussion with specific medication codes are a systemic change that has the potential to decline the number of statin overdose hospital visits in this demographic.

## Conclusions

As society witnesses a demographic landscape shift towards an aging populace, it becomes imperative to implement strategies that mitigate the risk of accidental ingestion primarily by children. Data show that drugs, such as atorvastatin, which are produced in attractive colors and shapes are more likely to be unintentionally consumed by young children. The peak incidence falls around the age of two, coinciding with the oral fixation developmental milestone is prevalent. The data is inconclusive regarding whether gender plays a role in the ingestion of statins. Although patients at such a young age may not be able to be effectively educated, there can be a discussion of safe prescription taking with the guardian. In this discussion, factors such as socioeconomic status and home environment should be considered by the physician. Within the realm of research, potential advances to find an effective antidote against statins to curb any harmful side effects that may come with overdose could reduce the length of emergency care visits for this cause. Recognizing and confronting this challenge proactively allows for the cultivation of a safer environment for children while remaining mindful of the dynamic requirements of an aging population.
